# Insulin Growth Factor Binding Protein 7 (IGFBP7)-Related Cancer and IGFBP3 and IGFBP7 Crosstalk

**DOI:** 10.3389/fonc.2020.00727

**Published:** 2020-05-15

**Authors:** Li Jin, Fan Shen, Michael Weinfeld, Consolato Sergi

**Affiliations:** ^1^Department of Laboratory Medicine, Shiyan Taihe Hospital, College of Biomedical Engineering, Hubei University of Medicine, Shiyan, China; ^2^Department of Laboratory Medicine and Pathology, University of Alberta, Edmonton, AB, Canada; ^3^Division of Experimental Oncology, Department of Oncology, Cross Cancer Institute, University of Alberta, Edmonton, AB, Canada; ^4^Department of Orthopedics, Tianyou Hospital, Wuhan University of Science and Technology, Wuhan, China; ^5^Key Laboratory of Fermentation Engineering, National “111” Center for Cellular Regulation and Molecular Pharmaceutics, Hubei University of Technology, Wuhan, China; ^6^Stollery Children's Hospital, University Alberta Hospital, Edmonton, AB, Canada

**Keywords:** cancer, growth factors, insulin, binding proteins, mechanism, cross-talk

## Abstract

The insulin/insulin-like growth factors (IGFs) have crucial tasks in the growth, differentiation, and proliferation of healthy and pernicious cells. They are involved in coordinated complexes, including receptors, ligands, binding proteins, and proteases. However, the systems can become dysregulated in tumorigenesis. Insulin-like growth factor-binding protein 7 (IGFBP7) is a protein belonging to the IGFBP superfamily (also termed GFBP-related proteins). Numerous studies have provided evidence that IGFBP3 and IGFBP7 are involved in a variety of cancers, including hepatocellular carcinoma (HCC), breast cancer, gastroesophageal cancer, colon cancer, prostate cancer, among many others. Still, very few suggest an interaction between these two molecules. In studying several cancer types in our laboratories, we found that both proteins share some crucial signaling pathways. The objective of this review is to present a comprehensive overview of the relationship between IGFBP7 and cancer, as well as highlighting IGFBP3 crosstalk with IGFBP7 reported in recent studies.

## Introduction

### IGF System

The insulin-like growth factor (IGF) signaling axis portrays an essential and pivotal role in cell growth, differentiation, and metabolic processes within the human body. It plays a central role in the development from gestation, continuing its role during life through the stimulation of cell proliferation and blocking programmed cell death (1, 2). The IGF system includes two complex coordinated growth factors (IGF-I and IGF-II), and three receptors (IGF-IR, IGF-IIR, and the insulin receptor, IR), six binding proteins (IGFBP-1 to 6) with high-affinity, a group of IGFBP proteases (kallikreins, cathepsins, and matrix metalloproteinases, MMPs), as well as several IGFBP-related proteins (IGFBP-rP 1 to 10) with low affinity (3, 4). Binding of IGF-1 and IGF-2 with their respective receptors has resulted in a better understanding of this complex machinery. It directs the activation of (1) Ras-Raf-mitogen-activated protein kinases (MAPK) signaling and (2) phosphatidylinositol-4,5-bisphosphate 3-kinase (PI3K)-AKT, through which the IGF axis controls cellular metabolism, tissue homeostasis, and, ultimately, cell survival (5, 6).

Several studies have demonstrated that during tumor progression, the influence of the insulin/IGF system on cancer cell behavior is primarily through the regulation of the epithelial-mesenchymal transition (EMT) program to obtain a malignant phenotype (7–10). The insulin/IGF system is likewise involved in cancer cell metabolism, resistance to cancer drugs, and cancer stem cell (CSC) phenotypes (11, 12), which emphasize the significance of this system in the monitoring networks of the development and progression of cancer.

### IGFBP System

There is evidence indicating that IGFBPs act as transporters of IGFs, lengthen their half-life, and regulate their access to their receptors. According to the distinct affinities to IGFs, IGFBPs are categorized into two groups—binding proteins (IGFBP1-6) with IGF high-affinity and IGFBP-related proteins (IGFBP-rP1-10) with IGF low-affinity. All the IGFBP genes consist of four exons, except *IGFBP3*, which contains five exons (including one exon, which is apparently not translated). The biological activity of IGFs not only depends on the interaction with IGFRs but also on the influence of the IGFBP family. In biological fluids, the IGFs are often connected with IGFBPs, being rare (<1%) in their free form (5, 13). The IGFBPs exist in precursor and mature forms. In terms of molecular structure, IGFBPs are broadly composed of three different domains - conserved N- and C-terminal domains, and a central domain. The N-terminal domain includes an IGFBP motif (GCGCCXXC) that has a significant function in IGF binding. The C-terminal domain is similarly highly conserved. It too plays a critical role in binding to IGFs. The central domain is often labeled as the “binding domain” in the literature. It is usually subjected to post-translational modifications, such as phosphorylation and glycosylation, to control IGFBP activities (3, 14). IGFBP-degrading proteases can also influence IGFBP activities, including several MMPs, primarily MMP-7 and MMP-9 (15–17). IGFBPs play critical functions in the activity of ligands via the modulation of their half-life. This modulation is obtained by hindering binding with the receptor or alternatively stimulating signaling activation through controlled release of the ligands (18, 19). The evidence also suggests that IGFBPs perform insulin/IGF-independent functions, including in the course of tumor progression (20). There are structural and functional similarities between IGFBP-rPs and IGFBPs, but IGFBP-rPs bind with a lower affinity for IGF-I (~100-fold lower) (21). IGFBPs are considered as holding a high affinity for binding to IGFs. This aspect may be due to the formation of a specific binding pocket in the IGFs, and it has been observed that both the N-terminus and, of course, the C-terminus bind to this crucial high-affinity site (22).

### IGFBP3

Like other IGFBPs, IGFBP3 is secreted by many types of cells. It is the major IGF carrier protein that acts in both IGF-independent and IGF-dependent modes in the bloodstream and is the most abundant circulating IGFBP (3, 23). Circulating IGFBP3 can form a ternary complex with IGFs and acid-labile subunit (ALS), a liver-derived growth hormone-regulated glycoprotein (20, 24). The ternary complex slows down IGFBP3 dissociation rates and extends the IGFBP3 circulating half-life (25). Among the other IGFBPs, only IGFBP-5 forms a similar ternary complex. These complexes preserve very high concentrations of IGFs in healthy adults. The IGFBP3 C-terminal domain, not only binds IGF but has additionally been shown to be necessary for communications with the ALS all through the IGFBP3 key region Lys 228–Arg 232 (22). IGFBP3 plays a significant role involving IGF binding and modulating the distinct biological activities of the IGFs, such as cellular proliferation, cell differentiation, increasing metabolism, and cell survival by regulating their access to the IGF-I receptor (20, 26–29). The affinity between IGFBP3 and IGF-1 is higher than that of IGF-1 and its receptor so that IGFBP3 can compete with IGF blocking the combination of IGF to their receptors. Consequently, IGFBP3 can inhibit cell proliferation and survival (20). Consistent with this, the loss of IGFBP3 expression and consequent de-repression of IGF1R signaling can result in learned resistance to the well-known EGFR tyrosine kinase inhibitor *gefitinib* (30). IGFBP3 also has IGF-independent antiproliferative and proapoptotic effects, including its interaction with nuclear hormone receptors such as the retinoid X receptor (31, 32) and the vitamin D receptor (33), the TGF β/SMAD (mothers against decapentaplegic) signaling pathways (34, 35), and the upregulation of apoptotic effectors (36). The Smad family comprises receptor-regulated (R-) Smads (Smad 1, 2, 3, 5, and 8), common (Co-) Smad 4, and inhibitory (I-) Smads 6 and 7 (37). In the Smad family, Smad 2 and Smad 3 are strongly activated in renal fibrosis in diabetic nephropathy (37).

The deactivation of several proteases regulates the IGF-dependent and IGF-independent activity of IGFBP3. According to the tissue type, IGFBP3 specific proteases seem to change. For example, in the quiescent epidermis, MMP-19 is a major IGFBP3 degrading MMP (38), while matrix metalloproteinase-7 seems to degrade IGFBP3 in tumor tissues, enabling IGF bioavailability. Precise data of co-incubation of the IGF-I/IGFBP3 complex with MMP-7 highlighted that such an experiment restores IGF-I-mediated IGF-IR phosphorylation and, at the same time, activates AKT in cancer cell lines. This data is outstanding because it indicates that MMP-7 proteolysis of IGFBP3 performs a critical function in synchronizing IGF-I bioavailability, thereby ultimately promoting cell survival (15).

### IGFBP7

**Figure 1:**
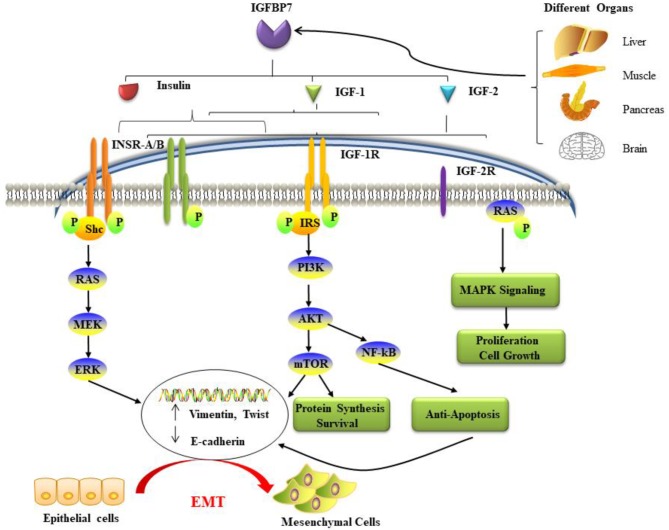
The potential mechanisms and pathways associated with IGFBP7 and cancer. IGFBP7 is expressed at both the protein and mRNA levels in most normal tissues, including the brain, liver, pancreas, and skeletal muscle, and is discharged into circulation. Insulin, IGF-1, and IGF-2 can bind to insulin receptors (INSR-A and INSR-B), IGF-2R has binding affinity only for IGF-2. Ligand activation of IGF-1R results in intrinsic tyrosine kinase phosphorylation. Also, it broadly activates (a crescent is placed backward with “backward” effect in the schema) three main (signaling) pathways: IRS-PI3K-AKT-mTOR signaling, Ras-MEK-ERK pathways, and Ras-MAPK signaling. The first two major pathways induce epithelial cells to lose their cell-cell adhesion and acquire the cellular identity of the mesenchymal phenotype. Loss of epithelial markers such as the cell adhesion molecule E-cadherin and the gain of Vimentin and other mesenchymal markers are considered hallmarks in the initiation and execution of EMT. Activation of the three pathways induces many different effects such as protein synthesis, proliferation, anti-apoptosis, cell survival, and growth. EMT: epithelial-mesenchymal transition; IGF: insulin-like growth factor; IGFBP7: IGF binding protein 7; IGF-1R: IGF-1 receptor; IGF-2R: IGF-2 receptor; INSR: insulin receptor; IRS: insulin receptor substrate; MAPK: mitogen-activated protein kinase; PI3K: phosphatidylinositol-4,5-bisphosphate 3-kinase.

IGFBP7 was the first component of IGFBP-related proteins to be discovered. Furthermore, it has been designated as insulin-like growth factor-binding protein-related protein-1 (IGFBP-rp1). This molecule has triggered an enormous interest in cell biology, because it is a secreted protein of a family of low-affinity IGFBPs termed IGFBP-rp1–10 (22, 39). It was initially named IGFBP7 because of its capability to attach IGFs through the N-terminal domain (40). IGFBP7 has been cloned from numerous kinds of cellular systems. Thus, it has acquired a variety of differing nomenclature, such as mac25, prostacyclin-stimulating factor (PSF), tumor adhesion factor (TAF), and angiomodulin (AGM). The *IGFBP7* gene has been mapped to chromosome 4q12. At the N-terminus of the IGFBP7 molecule, there is an IGFBP motif (GCGCCXXC) in a domain including 12 conserved amino acids (cysteines). The C-terminus of IGFBP7 differs substantially from the other IGFBPs because it lacks the conserved cysteines and in fact has only one cysteine (41). Also, while it has a 100-fold lower affinity for IGF-1 than the other IGFBPs, in contrast to the other family members it binds strongly to insulin, and essentially inhibits the phosphorylation of the insulin receptor (40, 42, 43). It has been suggested that the site that binds insulin could be at, or near, the IGF binding site (44) (Figure 1).

IGFBP7 possesses an IGF-independent activity (22). It shows strong homology with follistatin (also called activin-binding protein and encoded by the *FST* gene), although it lacks the C-terminus of follistatin (45). Like follistatin, IGFBP7 can bind to activin A, and consequently influence the growth-suppressing effects of the TGF-β superfamily. Expression of IGFBP7 has been shown to be upregulated in cells treated with TGF-β1 and retinoic acid (RA) (3, 46). IGFBP7 also binds to heparan sulfate on the cell surface, but this interaction may be affected by cleavage of IGFBP7 by the trypsin-like integral-membrane serine peptidase matriptase (47). Matriptase, which cleaves substrates with Arg or Lys at the P1 position, has been incriminated in invasion and metastasis of breast cancer (48–51). The action of proteolytic cleavage, specifically located at the N-terminus, including the heparin-binding motif, decreases heparin-binding and the occupancy of IGF-1R (48, 52, 53).

Furthermore, IGFBP7 was also discovered to bind to type IV collagen, initially by observing co-localization in the vascular basement membrane, and subsequently by direct measurement of attachment of radiolabeled IGFBP7 to extracellular matrix proteins (54). These authors also observed that IGFBP7 stimulated adhesion of human umbilical vein endothelial cells to type IV collagen substrate inducing morphological changes. St. Croix et al. (55) also found that IGFBP7 has a role in binding with type IV collagen. They showed that the *IGFBP7* expression in tumor-associated endothelium is much higher than in healthy endothelial cells. IGFBP7 may also be a potential tumor endothelial cell marker, as identified by serial analysis of gene expression (SAGE). In this study, IGFBP7 and type IV collagen are co-expressed and interact with each other (55).

*IGFBP7* mRNA is expressed in an extensive variety of human tissues, including liver, pancreas, small intestine, large intestine (gastrointestinal tract), as well as extra-gastrointestinal organs (brain, heart, spleen, kidney, placenta, lung, skeletal muscle, thymus, prostate, testis, ovary) as detected by northern blot hybridization (56). The mRNA expression of *IGFBP7* and, of course, its translation is regulated by IGF-I, TGF-β, and RA in epithelial cells of the prostatic gland (57). The immunohistochemical investigations performed on human prostatic tissues (normal) shows ubiquitous intense staining. Strong staining was also observed in the cilium of the respiratory system, epididymis, and fallopian tube. Most endothelial cells are positive, while fat cells, lymphocytes, and plasma cells are negative. *IGFBP7* expression was restricted to specific cell types in the adrenal gland, kidney, and skeletal muscle, intimating a possible specialized role in these organs (56). IGFBP7 was also measurable in most human body fluids such as a serum, urine, and cerebrospinal fluid as well as amniotic fluid of pregnant individuals (58).

In contrast to IGFBP3 and IGFBP5, IGFBP7 is not influenced by glycosylation or phosphorylation. Proteolytic processing can modulate the protein expression level of *IGFBP7*. IGFBP7 is differentiated from other IGFBPs through regulatory mechanisms at the RNA and DNA levels (3).

IGFBP 7 is not only detectable in several healthy human tissues and human biological fluids, but also a variety of diverse cancers. However, importantly, accumulating studies clarified that *IGFBP7* is up-regulated in some types of malignancies and down-regulated in others (Table 1). These entirely different manifestations suggest that IGFBP7 functions may be a “double-edged sword” in cancer cell proliferation, progression, and prognosis, displaying an ambiguous action as an oncogene or suppressor gene in distinct types of cancers. The potential mechanisms and pathways associated with IGFBP7 and cancer are summarized in Figure 1. Our group has studied this aspect for Sirt1 (59, 79). Moreover, IGFBP7 can change cell sensitivity to chemotherapeutic drugs (60, 80), which suggests a potential beneficial value in anticancer treatment. In Table 1, the relationship between IGFBP7 and several of the most common cancers is highlighted.

**Table 1:** IGFBP7 expression in various tumors.

**Cancer type**	**Expression status**	**Test methods**	**References**
Breast	Down-regulation	qRT-PCR	(57, 59)
	Down-regulation	IHC	(57, 58)
Gastric	Up-regulation	IHC, qRT-PCR	(60, 61)
	Down-regulation	IHC, qRT-PCR	(62)
Prostate	Down-regulation	Northern blot, ISH	(48, 63)
	Up-regulation	IHC	(20)
Hepatocellular	Down-regulation	IHC, qRT-PCR	(64)
Colorectal	Up-regulation	IHC, Northern blot, ELISA	(65–69)
	Down-regulation	IHC	(70)
Lung	Down-regulation	IHC, qRT-PCR, Northern blot	(52, 71, 72)
Esophageal	Up-regulation	IHC	(73, 74)
Melanoma	Down-regulation	IHC	(75)
Glioma	Down-regulation	qRT-PCR	(76, 77)
	Up-reguation	qRT-PCR, Western blot	(78)

*IHC, immunohistochemistry; ISH, in situ hybridization; qRT-PCR, quantitative reverse transcriptase-polymerase chain reaction; ELISA, enzyme-linked immunosorbent assay*.

## IGFBP7 With Epithelial Tumors

We will now give an outline of studies of IGFBP7 and their importance in malignant neoplasms, such as epithelial tumors and mesenchymal tumors.

### Breast Cancer

In the normal breast, IGFBP7 is identified in the epithelial layer of the breast ducts and lobules (56). Swisshelm et al. (46) found that the *IGFBP7* gene is overexpressed in human mammary epithelial cells in senescence. The same authors report that IGFBP7 is overexpressed in normal, growing mammary epithelial cells exposed to all-trans-RA or *fenretinide*, a synthetic retinoid. However, immunohistochemical studies have indicated that mammary carcinoma epithelium does not show, or only lightly shows, IGFBP7 staining. Intense staining of IGFBP7 is found in normal and benign breast tissues but at low or untraceable levels in non-invasive breast cancers (DCIS). It is lost in all the invasive malignancies investigated. IGFBP7 expression is correlated with breast cancer progression (61, 81, 82). Burger et al. (61) reported that IGFBP7 is expressed at much lower levels in metastatic neoplasms than in matched primary tumors, which was associated with a loss of heterozygosity (LOH) in 50% of the cases. These authors also found that serial transplantation of xenografted human primary tumors reduces the levels of *IGFBP7* mRNA at each transfer. Using qRT-PCR, Subramanian et al. (82) found that the expression of *IGFBP7* mRNA in patient groups with TNM1 and TNM2 breast cancer was much higher than the poorer prognosis TNM3 group. The IGFBP7 level is also associated with the differentiation and prognosis of patients. Low IGFBP7 rate had a significantly reduced survival, poor differentiation, and a higher tumor stage; in all examined breast cancer cases, 15% lacked IGFBP7 staining entirely, but 20% had weak staining, 32% intermediate and 33%, finally, showed strong staining (83). Over-expression of IGFBP7 in MDA-MB-468, a triple-negative breast cancer (TNBC) cell line, reduced cell division and migration through a specific inhibition of phosphorylation of MAPK (extracellular) signal-regulated kinase (ERK)-1/2 (81). The ability to reduce tumor growth also occurs *in vivo*. MDA-MB-231 is also a TNBC cell line. Utilizing this cell line and its more aggressive derivative cell line, 1833, Benatar et al. showed that treatment of TNBC cells with IGFBP7 causes cell cycle arrest and reduced cell growth, followed by induction of apoptosis or senescence, depending on the length of IGFBP7 exposure and dose (62). The xenografted tumor also shows decreased angiogenesis following tail-vein injection of recombinant IGFBP7, as demonstrated by significantly reduced CD34 and, most importantly, vascular endothelial growth factor (VEGF) expression. Zuo et al. (84) found that IGFBP7 inhibits MCF-7 cell proliferation and induces cellular senescence as a result of enhanced expression of p21 through a p53-independent pathway. p21Cip1, also known as cyclin-dependent kinase inhibitor 1 or CDK-interacting protein 1, is a cyclin-dependent kinase inhibitor that is able to inhibit all cyclin/CDK complexes The MCF-7 cells were arrested in G1 phase of the cell cycle. This setting adds to data generated by Wilson et al. (71) that indicated that IGFBP7 might act as a tumor suppressor by triggering senescence in breast cancer.

### Gastric Cancer

Some initial experiments have implied that *IGFBP7* is upregulated in gastric cancer patients matched to paired normal tissue based on immunohistochemistry and, quantitatively, on real-time polymerase chain reaction (qRT-PCR) (65, 66, 72, 85, 86). Takeno et al. (72) used a large-scale gene expression profiling and network analysis to identify genes accountable for tumorigenesis and tumor progression in patients affected with gastric cancer. These authors observed significant upregulation of *IGFBP7* in undifferentiated cancers matched to the differentiated ones, indicating that IGFBP7 may participate as a critical functional component in gastric cancer differentiation. A constructive theory was identified originating from a correlation between the IGFBP7 expression and the depth of tumor invasion, the presence and the number of metastatic lymph nodes, distant metastasis/recurrence, or pathological stage in the stomach cancer individuals (85). In marked contrast to these reports, the low expression of *IGFBP7* was also found to be associated with tumor progression and patients' poor survival in carcinoma of the stomach (67). Liu et al. suggest that the conflicting results may be caused by the differences in the tumor sites that were assessed in these studies. The contradictory data may be resolved by considering the non-homogeneous expression of *IGFBP7* (68) and that IGFBP7 signaling in stromal fibroblasts might override its tumor-suppressor role in epithelial cells.

### Lung Tumors

Healthy lung tissues show high IGFBP7 expression, which was investigated at the protein and mRNA levels. IGFBP7 was particularly evident in the small airway bronchial epithelial cells and the cytoplasm of lung bronchial epithelial cells, despite low expression of IGFBP7 in primary lung cancer tissues (69). Okamura et al. found that IGFBP7 expression was reduced in human primary and metastatic pulmonary carcinoma compared with healthy lung tissues (80). Expression of the *IGFBP7* gene did not diverge significantly between tumors of different types, different stages (including tumor size and lymph node status), and grading (69, 87). DNA hypermethylation resulted in lower *IGFBP7* expression for both lung cancer cell lines and primary lung tumors (87). The same report also indicated that most of the lung cancer cell lines showed down-regulation of *IGFBP7* compared to normal tissues, and there was no difference in the expression pattern of IGFBP7 between small cell lung cancer (SCLC) cell lines and non-small cell lung cancer cell lines (NSCLC) (87). The qRT-PCR and immunohistochemistry results were congruent with the data from northern blot and western blot analysis (69). IGFBP7-positive transfectants of the H2170 cell line displayed remarkably decreased colony-forming ability in soft agar (*in vitro* cellular transformation technique), suppression of tumor growth rate in nude mice (genetic mutation harboring strain with deteriorated or absent thymus and subsequent inhibited immune system), and increased cellular apoptosis (programmed cell death), as well as activated caspase-3 expression level (69). A high serum level of IGFBP7 correlated with positive nodal status and could be a valuable biomarker to identify patients with poor outcomes in NSCLC. Another report suggested that downregulation of *IGFBP7* rendered lung cancer cells resistant to cisplatin and altered the sensitivity to cisplatin-based anticancer therapy (80). A similar study reported that methylation and transcriptional silencing of specific genes are associated with the increase of cisplatin resistance (88). Also, Creighton et al. (89) showed that *IGFBP7* was up-regulated in tumor xenografts created by the implantation of human lung adenocarcinoma (A549) cells in experimental animals.

### Colorectal Cancer

Initial studies have suggested that IGFBP7 expression is drastically up-regulated in colorectal cancer (CRC) tissues compared with their paired non-cancerous tissues (70, 90). Follow-up studies reported similar data (91–93). Shao et al. observed that the upregulation of IGFBP7 is universal in colorectal cancer, especially in subjects affected with type II diabetes mellitus (DM). Ruan et al. (63, 93, 94) indicated that IGFBP7 is robustly expressed in colorectal carcinoma of low-grade type while it is weakly expressed in the high-grade type of colorectal carcinoma. However, this study's conclusion is in conflict with the finding of Adachi et al. who discovered *IGFBP7* expression was correlated with a reduced overall outcome (90). *IGFBP7* expression was mostly increased in CRC patients with Dukes' stages B and C compared to Dukes' stage A. Their findings suggest that plasma IGFBP7 levels do not reflect the local level of IGFBP7 in neoplastic tissue, and there is a significant difference regarding stage (of the neoplastic disease) and *IGFBP7* expression. Thus, the expression of *IGFBP7* may reflect disease progression. Also, a gradient of IGFBP7 immunohistochemistry staining is found along the axis of the crypt in human normal colonic epithelium, with much higher IGFBP7 expression in the differentiated epithelial cells at the surface compared to the lower expression at the crypt base (63). During Caco2 cell differentiation induced by sodium butyrate, IGFBP7 expression increased, and the expression showed a significant relationship with alkaline phosphatase (AKP) activity.

These findings suggested that IGFBP7 is a potential pivotal molecule for colon cancer differentiation. Two studies indicated that IGFBP7 has a unique function in inhibiting epithelial-mesenchymal transition (EMT) and metastasis via a TGF-β/Smad-dependent pathway (63, 95). However, down-regulation of IGFBP7 expression was found in colorectal tissues from a cohort of the Caucasian population (96). This conclusion is contradictory to some other reports, and the difference may be related to ethnic differences. Lin et al. have reported that IGFBP7 expression is negatively correlated with its methylation status in CRC (97). The hypermethylation state of the CpG islands of IGFBP7 is responsible for its down-regulation. They also described that DNA methylation is one of the mechanisms that regulates the expression of IGFBP7. The expression of IGFBP7 is at a low level or lost in CRC cell lines (64, 98). In an *in vitro* study, transfecting exogenous IGFBP7 cDNA into CRC cells induced cell growth inhibition, cell cycle arrest, a decrease in soft agar colony formation, and programmed cell death (93). Sato et al. have shown that overexpression of IGFBP7 in neoplastic CRC cells with an epithelial phenotype such as DLD-1 reduces anchorage-independent colony formation and xenograft tumor growth (64). *IGFBP7* expression in tumor-associated fibroblasts can also stimulate colony formation when neoplastic epithelial cells are co-cultured with IGFBP7-expressing cancer-associated fibroblasts by secondary (paracrine) tumor-stroma interactions (68). In addition to its potential function as a tumor suppressor, Georges et al. demonstrated that IGFBP7 might have supplementary properties, which become obvious only in the broad framework of cancer progression, staging advancement, and metastasis formation (99). Recently, Li et al. (100) compared the expression of IGFBP7 in primary CRC with that in matched tissues arising from the liver metastases. They also investigated mechanistic aspects of IGFBP7 by examining the consequences of IGFBP7 knockdown and overexpression on the expression of EMT-associated proteins, such as E-cadherin, N-cadherin, and vimentin protein. These authors found that *IGFBP7* is significantly downregulated in liver metastasis of patients with CRC. Also, the authors found that IGFBP7 overexpression influences the expression of EMT-associated genes, such as E-cadherin, thereby regulating the metastatic behavior of CRC cells. This data is paramount, highlighting the biological and clinical significance of IGFBP7 in CRC, although the role of IGFBP7 in CRC-associated liver metastasis requires further investigation. These intriguing findings support the conclusion put forward by Georges' et al. that “IGFBP3 and 7 cannot be simply assigned to the group of tumor suppressors, but have additional properties, which become evident only in the context of cancer progression and metastasis formation” (99).

### Prostate Cancer

Several previous studies have detailed that *IGFBP7* gene expression is significantly diminished in prostate carcinoma based on RT-PCR, northern blot, and *in situ* hybridization (ISH) (57, 101). Hwa et al. observed a progressive decrease of detectable IGFBP7 expression going from healthy cells of the glandular epithelium of the prostate to malignant prostate epithelial cells (57). This study also described that protein and mRNA expression of IGFBP7 is regulated by IGF-I, TGF-β, and RA in prostatic epithelial cells. Mutaguchi et al. revealed that inactivation of IGFBP7 occurs through CpG methylation, and the tumor-suppressive activity of IGFBP7 relies on the induction of programmed cell death in an IGF-I independent manner in cancer of the prostatic gland (101). Moreover, Tennant et al. generated similar data confirming that IGFBP7 is detectable in the normal prostatic epithelium but progressively decreases when the prostatic epithelium degenerates toward malignancy (73). However, these findings have been challenged by Degeorges et al. who reported that prostatic intraepithelial neoplasia (PIN), prostatic adenocarcinoma, and prostatic cancer metastases express IGFBP7 with no differential based on stage or grade.

In contrast, secretory epithelium of the normal prostatic gland and benign prostatic hyperplasia do not usually express this molecule (102). Discordant results in prostatic gland research are unfortunately not uncommon, and even the classification of prostatic neoplasia has been changed several times in the last two decades. Moreover, non-neoplastic conditions that mimic prostate cancer have been better identified only in the previous decade. The observed differences in IGFBP7 expression might be caused by the different sources of prostatic epithelial cells. It seems incontrovertible that aberrant *IGFBP7* promoter hypermethylation and simultaneous *IGFBP7* gene silencing occurs in prostatic cancer cell lines, and the rate of *IGFBP7* hypermethylation is significantly higher in cancer of the prostatic gland (60%) and high grade prostatic intraepithelial neoplasia (40%) than that in benign prostatic hyperplasia (15%) and histologically benign prostatic epithelium adjacent to the tumor (0%) (103). The results also suggest that the CpG methylation of *IGFBP7* may be a unique biomarker of prostate cancer and pre-invasive neoplasms. Both IGFBP7 mRNA and protein expression are drastically increased during senescence of the normal epithelium of the prostatic gland (74), which supports the role of IGFBP7 as a tumor suppressor. The re-expression of IGFBP7 in a human prostate cancer cell line M12 led to a lengthening of cell doubling time, a decline in colony formation in soft agar, significant alteration in epithelial morphology, enhanced propensity to undergo apoptosis, and, finally, a reduction in the number and size of tumor formation *in vivo* (104). Another study suggested that over-expression of *IGFBP7* in M12 cells alters cell cycle kinetics by delaying the cells in the G1 phase of the cell cycle (103). They also reported that IGFBP7 expression in the M12 cell line results in expanded sensitivity to programmed cell death in agreement with the earlier study (104). Tennant et al. showed a significant inverse relationship between IGFBP7 expression levels and proliferation in the LuCaP 23.12 prostate tumor xenografts (73). The above findings suggest that IGFBP7 plays a pivotal role in prostate cancer tumor suppression and senescence.

### Hepatocellular Cancer

The principal source of IGFBPs is the liver, and IGFBP7 mRNA is expressed in healthy human liver (76). *IGFBP7* gene expression is clearly significantly down-regulated in human HCC tumor samples and HCC cell lines compared with healthy hepatocytes. It is inversely correlated with the clinical stages and histopathological grades of HCC (77). In each stage, *IGFBP7* expression was remarkably much lower in the poorly differentiated type of HCC compared with moderately differentiated HCC. Expression of *IGFBP7* is considerably downregulated by elevated astrocyte gene-1 (AEG-1), a novel oncogene that is overexpressed in 90% of the patients affected with HCC (73). This discovery suggests that *IGFBP7* downregulation may be needed to mediate the tumor-promoting function of *AEG-1*. Chen et al. (77) have found that stable overexpression of *IGFBP7* in HepG3 cells that artificially overexpressed AEG-1 led to a marked decrease in proliferation and colony formation, and induced senescence. They also found that there is a full deletion of the *IGFBP7* gene in some patients. Inhibition of angiogenesis, as evidenced by a decline in CD31 staining, was shown to be a major factor in the marked inhibition of tumor growth resulting from IGFBP7 overexpression in the HepG3-AEG-1 model system. Downregulation of IGFBP7 expression was connected with hypermethylation of the CpG islands in the *IGFBP7* promoter in murine SV40T/t antigen-induced hepato-carcinogenesis and human HBV-related HCC (78, 105). Li et al. found that the frequency of serum *IGFBP7* promoter methylation is significantly higher in HCC patients than the controls (105). This study also indicated that serum *IGFBP7* methylation is as sensitive as alpha-fetoprotein (AFP), a common malignancy biomarker extremely useful in the diagnosis of HCC.

Moreover, employing a combination of *IGFBP7* gene promoter methylation and AFP level can improve the sensitivity of AFP in the diagnosis of HCC. New research indicates that IGFBP7 can suppress not only cancer cells but also modulate the tumor microenvironment, and this double effect may have a lasting impact on inhibiting both primary tumors and distant metastasis (42). Xenografts without IGFBP7 display increased spontaneous tumorigenesis without affecting healthy development, increased proliferation, reduced senescence, and activation of the IGF1 signaling pathway. Conversely, the overexpression of *IGFBP7* decreases tumor growth by activating an antitumor immune response (2). Forced overexpression of *IGFBP7* in an AEG-1–overexpressing HCC cell model caused an inhibition of *in vitro* growth and, simultaneously, induced senescence, and extremely suppressed *in vivo* growth in nude mice that revealed inhibition of angiogenesis (77).

### Esophageal Cancer

Kashyap et al. have produced several reports regarding *IGFBP7* expression in esophageal carcinomas, including Barrett carcinoma, esophageal adenocarcinoma (EAC), and squamous cell carcinoma of the esophagus (ESCC) (106–108). These studies have shown up-regulation and more than 4-fold over-expression of IGFBP7 in Barrett metaplasia and EAC as related to healthy tissues. Also, they examined IGFBP7 expression in normal tissues and ESCC-derived tissue sections using immunohistochemistry. They found that more than 90% of ESCC cases were openly positive with identification in both the cytoplasm and nucleus in most of the histological tissue sections (106).

Furthermore, there is a significant increase in *IGFBP7* expression in EAC compared to healthy tissues (109), although these authors reported that there was a broad range of expression from low to high. Kim et al. have found consistent results showing that the expression of IGFBP7 was significantly increased in neoplasms with stage 3 compared with stage 0 neoplasms (75). DNA methylation of the *IGFBP7* promoter resulted in an association with transcriptional silencing in esophageal adenocarcinoma and Barrett carcinoma cell lines. Definitely, it was a common feature in Barrett and EAC tissues and correlated with the silencing of protein expression. The low IGFBP7 expression was discovered to be related to a more prolonged survival of patients harboring an EAC (74).

## IGFBP7 With Mesenchymal Tumors

### Glioma

Tian et al. found that IGFBP7 mRNA levels were substantially decreased in human glioma in comparison to healthy brain tissue (110) and that low levels of IGFBP7 mRNA were associated with larger tumor size, and patients with higher IGFBP7 expression had longer overall survival. Pen et al. (111) described that IGFBP7 acts as a tumor suppressor by lowering glioblastoma cell growth and inducing senescence, although not programmed cell death (111). Contrary to these findings, Jiang et al. found that IGFBP7 was highly overexpressed in glioma tissues compared to healthy brain tissues using RT-PCR and western blot. Furthermore, IGFBP7 expression correlated well with tumor grade and low overall glioma patient survival (112). This group also found that IGFBP7 mediates glioma cell growth and cell migration through the regulation of AKT and ERK1/2 activation (112). Other reports claimed that IGFBP7 was also robustly expressed in glioblastoma blood vessels and could induce the formation of “capillary-like tubes” by endothelial cells of the brain (113–116). These findings indicate that IGFBP7 stimulates glioma cell progression, and this aspect correlates with vessel formation. There are multiple grades of glioma with grade IV being the most malignant. Glioblastoma multiforme (GBM), which corresponds to grade IV, is the most aggressive and very infiltrative, and several mechanisms have been suggested for why the marked formation of blood vessels observed in this kind of glioma is associated with poor survival.

### Melanoma

A major study found that *IGFBP7* gene expression is missing in primary melanomas bearing an activating mutation of the *B-Raf* gene (*BRAF*) but apparently not in primary melanomas with wild-type BRAF (117). Its silencing is due to promoter hypermethylation. Also, the expression of *IGFBP7* in melanoma cells containing the *BRAF* mutation can induce cell senescence and apoptosis. Reinstatement of IGFBP7 function utilizing the addition of recombinant IGFBP7 (rIGFBP7) causes programmed cell death in BRAF-positive cell lines derived from human melanoma, and systemically administered rIGFBP7 also has the capacity to suppress the growth of BRAF-positive tumors in xenograft mouse models (118). The pronounced reduction of IGFBP7 expression in metastatic samples indicates that during the development of melanoma, there is a substantial selection against IGFBP7 expression. This finding suggests that *IGFBP7* acts as a melanoma tumor suppressor gene, which can inhibit BRAF-MEK-ERK signaling. It can efficiently induce programmed cell death in BRAF-positive melanoma cell lines (118). However, Scurr et al. concluded that BRAF mutational status has no relationship with the loss of IGFBP7 in melanoma (119). Employing intratumoral injection of a plasmid carrying IGFBP7 cDNA, Chen et al. found that melanoma growth in mice could be inhibited, due to the induction of apoptosis and the reduction in vascular endothelial growth factor (VEGF) expression (120). This new finding implies that IGFBP7 may have a potential therapeutic role in curing melanoma.

## IGFBP3 - IGFBP7 Crosstalk

**Figure 2:**
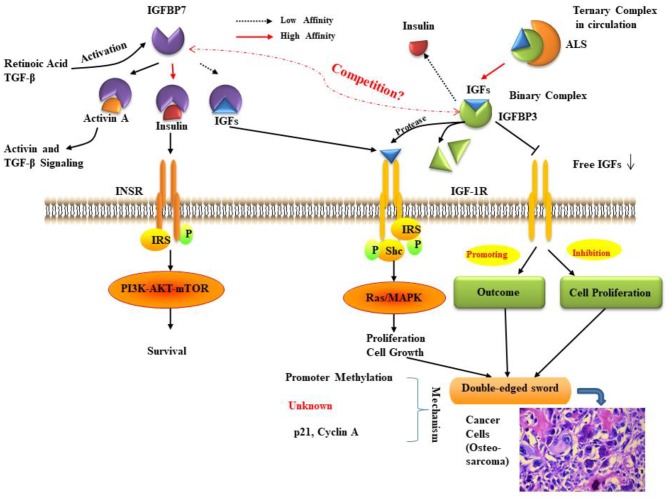
The relationship between IGFBP3 and IGFBP7. IGF bioavailability is determined by sequestration in (ternary) complexes with IGFBP3 and ALS in the circulation, or in (binary) complexes with IGFBP3 in the cell environment. IGFBP7 binding partners are insulin, IGF-I, and activin, the levels of which can be controlled by retinoic acid and TGF-β. The function of IGFBP7 and IGFBP3 may be a double-edged sword in cancer proliferation, prognosis, and survival. The molecular mechanisms for IGFBP7 and IGFBP3 suppression of cancer remain to be fully elucidated, but some studies indicate that the primary mechanisms involve promoter methylation and the regulation of p21. ALS: Acid labile subunit; IGF: insulin-like growth factor; IGFBP7: IGF binding protein 7; IGF-1R: IGF-1 receptor; INSR: insulin receptor; IRS: insulin receptor substrate; MAPK: mitogen-activated protein kinase; mTOR: mammalian target of rapamycin; Shc: Src homology 2 domain-containing; TGF: transforming growth factor. As cancer cells are shown (histological inset), osteosarcoma cells show anaplastic cytological features associated with osteoid formation.

The IGFBPs are a family of homologous proteins as indicated early in this review. They have co-developed with the IGFs (121). IGFBP3 and IGFBP7 are the unique members of the IGFBP superfamily that regulate the bioavailability of insulin and IGFs and have a similar structure and functions. IGFBP3 has a very high affinity for IGFs. In fact, it can transport >75% of serum IGF-I and -II, while its affinity for insulin is very low. Conversely, IGFBP7 attaches to insulin with a very high affinity (500-fold more elevated compared to other IGFBPs) but has a low affinity for IGF-I and -II protein (22). In the last few years, there has been an increasing number of studies and findings regarding the roles and mechanisms of IGFBP3 and IGFBP7, and their relationships with many kinds of diseases, especially in different cancer types. Some of these studies pay attention to the impact of IGFBP3/IGFBP7 interaction. Here we have reviewed the potential relationship of these two proteins (Figure 2) and their critical role in several kinds of common cancers and the development of treatments for cancers.

Several reports have described that the binding proteins have the ability to inhibit cell proliferation and increase apoptosis in neoplastic cells (2, 4, 20, 122). IGFBP3 has been proven to inhibit cell proliferation in breast, lung, colon, prostate, and bone tumor cells and some cell lines to reduce tumor cell growth (2, 3, 22, 27, 28). Coherent with these data, p53, and growth inhibitory agents, including RA, TGF-β, and anti-estrogens, can give rise to increased expression of IGFBP3 (20, 123). IGFBP7 can regulate the growth-suppressing effects of the TGF-β superfamily, and similarly the expression of IGFBP7 can be upregulated by cellular treatment with TGF-β1 and RA (46, 57, 74). IGFBP7 also plays a role as a tumor suppressor in the liver, urinary bladder, cervix uteri, lung, esophagus, thyroid, as well as head and neck cancer (42, 45, 46, 57, 74). It is a potentially pivotal molecule whose expression correlates with cancer differentiation (72, 95). Also, the expression of IGFBP3 and IGFBP7 is frequently reduced in several kinds of cancers, and this decrease is usually caused by promoter methylation (98, 124). The promoter of *IGFBP3* is hypermethylated in neoplasms of kidney, ovary, liver, stomach, large intestine, breast, and mesothelium (124–127). Another report described that DNA methylation and transcriptional silencing of specific genes are correlated with the expression of IGFBP7 in breast, lung, liver, prostate, esophageal, CRC cell lines (46, 64, 74, 78, 89, 98, 105, 109). Li et al. reported that the serum level of *IGFBP7* methylation is as sensitive as AFP in the diagnosis of HCC (105). Treatment with 5-aza-dC decreased expression of IGFBP3 in HCC cell lines (127) and IGFBP7 in CRC cell lines (97). Expression profiling identified three pathways altered in cellular immortalization of the p53 mutated skin fibroblast cell lines, MDAH041 and MDAH087: interferon, cell cycle, and cytoskeleton (128). Expression of IGFBP3 or IGFBP7 in these cells inhibited colony formation at low cell density. This finding is consistent with Wilson et al. who found that over-expression of IGFBP7 in MCF-7 breast cancer cells inhibits cell proliferation and induces cellular senescence (71). Georges et al. also described that the silencing of *IGFBP3* and *IGFBP7* in CRC cell lines lowered cell proliferation, colony formation, and, in the case of IGFBP3, also reduced cell migration (99). Increasing levels of IGFBP3 and IGFBP7 can reduce the levels of free IGFs, which in turn results in decreased binding of IGF to IGF-IR and IGF-IIR, leading to inhibition of cell growth and proliferation and potential activation of apoptosis (128). Moreover, the *IGFBP3* and *IGFBP7* genes are tightly regulated by their microenvironment. It seems that the stable expression of IGFBP3 and IGFBP7 is critical for many cellular functions because the regulation of these genes has been associated with malignant properties (78, 129, 130). The current data are probably limited, but it should be emphasized that additional properties of these two molecules in the context of cancer progression and metastasis will become evident in the next few years (116). The major attributes of IGFBPs that could enhance their abilities to regulate IGF actions at cytologic and extracellular levels include ternary complex formation, nuclear localization and transactivation, spatio-temporal expression pattern, and interaction with the cell surface and extracellular proteins (131). Recently, tumor microenvironment in cancer formation has been an object of intense investigation. Considering the vast field, we may mention some of the most recent proteo-transcriptomics data with a focus on ovarian cancer. A key feature of this disease is its unique microenvironment involving tumor cell spheroids (TU), tumor-associated T cells (TAT), and tumor-associated-macrophages (TAM) that promote cancer progression, chemoresistance, and immunosuppression. Recently, Worzfeld et al. presented data accrued from global, cell type-specific, parallel proteomic and transcriptomic analyses of tumor cell spheroids, TAT and TAM in the peritoneal microenvironment of high grade serous ovarian adenocarcinoma (HGSOC) (132). These authors identified several intercellular signaling pathways responsible for driving cancer cell adhesion, invasion and metastasis and immunosurveillance, all of which seem to be strongly associated with patient survival. CD163^low^ cells produce signaling molecules associated with favorable clinical outcome due to recruitment and activation of T cells (CXCL9–11, IL-15) and up-regulation of interferon signaling in TAM. Conversely, CD163^high^ cells secrete proteins promoting cancer cell stemness and angiogenesis. Both TAM and TU also release factors such as ECM remodeling proteins that reprogram immune cells to be immunosuppressed, and, therefore potentially, *pro*-tumorigenic (132).

## Discussion

Over the last decades, many laboratories have confirmed that IGFs play an essential and pivotal role in cell growth, differentiation, and metabolic processes within the human body (1, 2). It seems clear that IGFBP7 and IGFBP3 are unique and critical members of the IGF signaling axis. IGFBP3 is the most abundant circulating IGFBP and a major IGF carrier protein (3, 23), while IGFBP7 is a high-affinity insulin-binding protein. In different types of cancers, IGFBP7 plays an inhibitory role by curbing proliferation and inducing apoptosis and senescence. However, there are also several conflicting findings and different conclusions, which clearly demonstrate that IGFBP7 is a key player responsible for tumorigenesis and tumor progression in patients affected by gastric cancer (72). These entirely different opinions suggest that IGFBP7 functions may be a “double-edged sword” in cancer proliferation, progression, and prognosis. IGFBP3 can function as a tumor suppressor and is downregulated in some neoplastic tissues, but it nevertheless shows overexpression in association with markers of poor outcome in some tumor types (133–135). Especially in breast cancer, the opposite result is seen in some studies (133, 134). IGFBP3, therefore, appears to elicit a 2-fold response in tumors. This conclusion also pertains to the expression of IGFBP7 in prostate and colorectal cancer (57, 90, 96, 102). The expression of IGFBP3 and IGFBP7 is often reduced in several kinds of cancers. This decline is usually caused by promoter methylation transcriptional silencing of specific genes (46, 124). The reasons for the observed increases in IGFBP3 and IGFBP7 expression remain unclear at present. The contradictory data suggest that IGFBP3 and IGFBP7 might be antiapoptotic or proapoptotic, depending on the cellular environments. Shao et al. have reported relatively high expression of *IGFBP7* in most colorectal cancer patients with type II diabetes mellitus (DM) (91). Furthermore, the expression level appeared to be significantly related to the level of fasting glucose in these patients. Also, IGFBP7 is found to be increased in type 2 DM patients receiving no pharmacological therapy (136). Although there is limited understanding of the detailed molecular mechanisms so far, there are several papers that have provided evidence for mechanisms for IGFBP7 involvement in suppression of cancer, one of which is the critical mechanism of inactivation of IGFBP7 expression by aberrant hypermethylation (93); a second mechanism has been proposed to be via regulation of p21 and cyclin A/CDK-2 activity and resulting induction of apoptosis (137). The upregulation of IGFBP7 may be triggered by alterations in the gene structure or abnormalities of upstream regulatory mechanisms (91). The serum level of IGFBP7 methylation is as sensitive as AFP in the diagnosis of HCC (105). This finding indicates that IGFBP7 may be a potential diagnostic biomarker in cancers. Further investigations are required to identify the complex biological role and detail molecular mechanisms of each IGFBP in different cancer types and their relationship with insulin and plasma glucose to elucidate the relationship between cancers and type II DM (29, 59, 138, 139). In consideration of the obesity and diabetes pandemics, the re-expression of these genes and genes associated with growth factors in complex signaling pathways may boost a rational strategy for blocking cancer formation at an early stage.

## Author Contributions

LJ and CS revised the topic in the literature systematically. LJ wrote the first draft of the manuscript. FS revised the statistics of several studies. CS secured funding. MW revised critically the draft. LJ, FS, MW, and CS reviewed and approved the final version of the manuscript.

## Conflict of Interest

The authors declare that the research was conducted in the absence of any commercial or financial relationships that could be construed as a potential conflict of interest.
